# Plain Radiographs Can Safely Be Used to Measure and Follow Up on Tibial Component Alignment in Unicondylar Knee Replacement: A Correlation Study With CT Scans

**DOI:** 10.7759/cureus.16902

**Published:** 2021-08-05

**Authors:** Enejd Veizi, Nurdan Çay, Şahan Güven, Ali Şahin, Ahmet Fırat, Kasım Kılıçarslan

**Affiliations:** 1 Orthopedics and Traumatology, Ankara City Hospital, Ankara, TUR; 2 Radiology, Ankara City Hospital, Ankara, TUR

**Keywords:** unicondylar knee arthoplasty, oxford knee, knee alignment, correlational study, arthroplasty

## Abstract

Background and objective

Unicondylar knee replacement (UKR) is one of the most frequently performed arthroplastic operations worldwide. Migration and subsidence regarding the tibial component of UKR is a well-known phenomenon. In this study, we aimed to analyze whether plain radiographs are a reliable means to measure the true coronal and sagittal alignment of the UKR’s tibial component.

Methods

Patients undergoing a UKR procedure at our center between December 2020 and March 2011 were eligible for this study. Inclusion criteria were as follows: the presence of well-aligned standard and reproducible anteroposterior and lateral X-rays taken one week before or after a low-radiation artifact-reduced CT scan. Sixty-six knees were included in the study. Coronal and sagittal alignment of the tibial component was measured in a standard manner by two observers on both X-rays and CT scans. A correlation analysis was performed, and the margin of error was established.

Results

Intra-observer reliability was high among the two observers whether for X-ray or CT scan measurements [intraclass correlation coefficient (ICC): >0.900]. On the other hand, coronal plane measurements had lower inter-observer ICC values on both X-rays and CT scans while reliability on the sagittal plane was higher. There was a high correlation between radiographic measurements on X-rays and CT scans on both planes.

Conclusion

Even though the measurements on plain radiographs were slightly different from the ones obtained from CT scans, the correlation between them was very strong. Caution should be exercised when measuring the coronal alignment of the tibial implant on X-rays since it is more frequently affected by rotational misalignment.

## Introduction

Unicondylar knee replacement (UKR) is one of the most frequently performed arthroplastic operations worldwide, and the number of procedures has been on the rise in recent years [1-4]. When compared to total knee arthroplasty, the procedure is relatively safe, leads to less blood loss, and has shown good clinical and functional results with high rates of survival [5].

Migration and subsidence with regard to the tibial component of UKR, whether cemented or cementless, is a well-known phenomenon that has been reported since the emergence of the first implant designs [6]. Often leading to premature revisions, especially in cemented cases [7,8], migration and subsidence patterns have been previously described in the literature [9,10]. The general consensus is that the component tends to subside more in the early postoperative months and then remain stable with good results in the long term.

Radiostereometric analysis (RSA) has often been cited as the gold standard procedure to measure migration and subsidence, but it requires special equipment and extra, intraoperatively inserted tantalum landmarks, which is not possible in every orthopedic center [9]. CT and plain radiographs have also been used to measure subsidence and migration, but their reliability has been put into question due to the differences in the techniques used, lack of validation studies, and unknown correlation between the real values and the measured ones [11].

In this study, we examine the relationship between the coronal and sagittal alignment measurements of the tibial component of UKR implants measured on plain radiographs and on CT scans. We aim to analyze whether the plain radiograph, taken postoperatively in a standard manner and reproducible in every center worldwide, is a reliable means to measure the true coronal and sagittal alignment of the UKR tibial component. We hypothesize that although the measurements made on plain radiographs would be slightly different from the ones made on the CT scan, a strong correlation would still exist between the two.

## Materials and methods

Patients

All patients who underwent a UKR procedure at our orthopedic center between December 2020 and March 2011 were prospectively informed of the study design before surgery. Both mobile-bearing and fixed-bearing implants, whether cemented or uncemented, were selected for the study. Patients electing to undergo a CT scan postoperatively or during follow-up were included in this study. Inclusion criteria were the presence of well-aligned standard and reproducible anteroposterior and lateral X-rays taken one week before or after a low-radiation artifact-reduced CT scan of the operated knee. Direct standing radiographs were used for the study with an X-ray plate placed on the backside of the knee for anteroposterior views and on the inner side of the knee for lateral views. The plate included 20 cm of the upper tibia on the film and the X-ray source was situated one meter from the knee. In optimal anteroposterior views, the femoral and tibial condyles had to be symmetrical with the head of the fibula slightly superimposed with the lateral tibial condyle. For an optimal lateral view, the patient had to be standing with the knee flexed to 20 degrees, and superimposition of the femoral condyles was required.

This study was approved by our local Ethics Committee (number E2-21-30) on 13/01/2021. Informed consent was obtained from all patients included in the study.

We also researched our center’s database for patients with a UKR who had undergone a CT scan independently from our study and for different reasons (alignment/loosening analysis, intraarticular foreign body, mobile-bearing dislocations, before revision surgery, etc.). These patients were only included if they had well-aligned anteroposterior and lateral X-rays taken on the same day of the CT scan or in-between a week.

Exclusion criteria were the lack of well-aligned radiographic views despite the presence of a CT scan, CT scans not taken in an artifact-reduced mode, CT scans and X-rays taken for implant breakage or fracture cases, and cases who had their X-rays or CT scans taken on other orthopedic centers.

**Figure 1:**
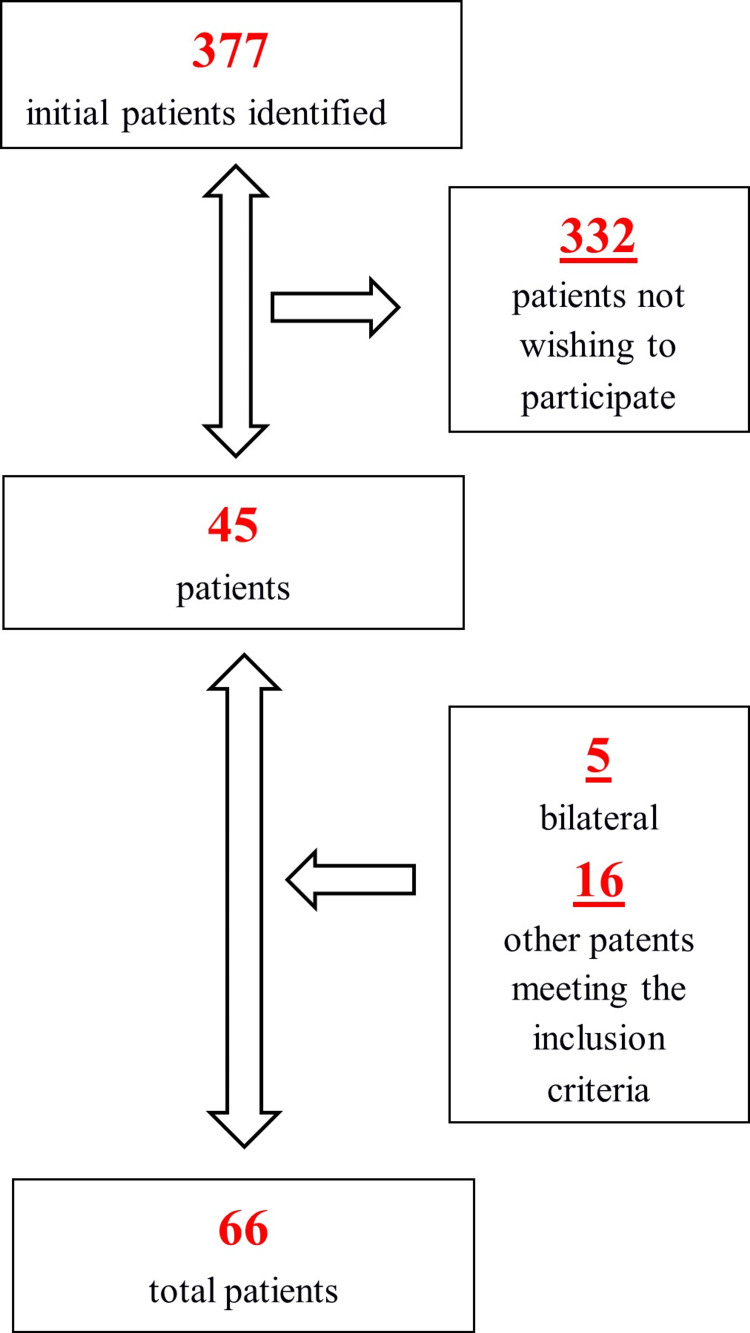
Diagram depicting inclusion/exclusion criteria and patient selection for the current study

A total of 377 patients underwent a UKR procedure at our center between the mentioned dates. Only 45 patients chose to be included in the study. Five of these patients underwent bilateral procedures. Our retrospective database search detected 16 other patients with a well-aligned X-ray and CT scan who met the inclusion criteria of the study. Finally, a total of 66 knees with their X-rays and CT scans were included in this study (Figure 1).

Radiographic analysis

**Figure 2:**
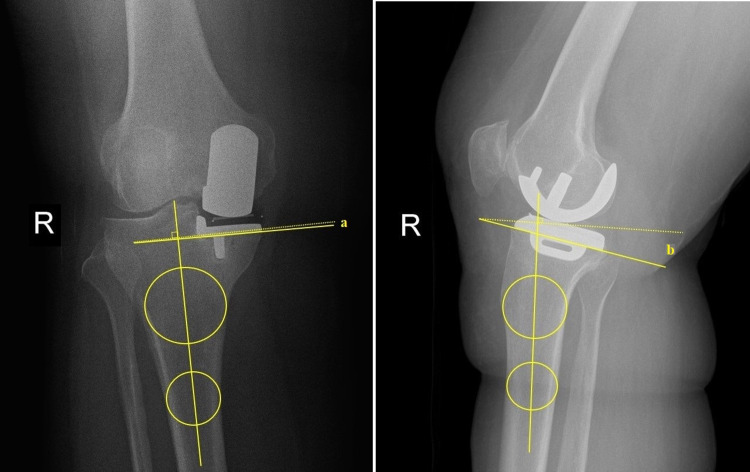
Standard anteroposterior and lateral views of an operated knee On the anteroposterior view, the tibial implant has to be perpendicular to the beam, thereby allowing a clear view of the joint space, and on the lateral radiographs, the femoral component has to be aligned to be perpendicular to the beam with the femoral condyles superimposed, and the tibial implant should be parallel to the horizontal plane. The coronal alignment of the tibial implants was measured with two circles drawn at a distance of 5 and 10 cm from the joint line and tangent to the medial and lateral cortex. A line passing between the midpoints of these circles and a line passing just underneath the tibial implant was used to measure coronal alignment (angle ‘a’). The sagittal alignment (slope) was measured with a line connecting midpoints of two intracortical circles at 5 and 10 cm distal to the knee joint and a line drawn just beneath the tibial implant (angle ‘b’).

Routine anteroposterior and lateral knee radiographs of the operated knees were taken on every follow-up. Radiographs that were taken preferably the same day or within a week before or after the CT scan were aligned in the coronal and the sagittal plane. On anteroposterior views, the tibial implant had to be perpendicular to the beam, allowing a clear view of the joint space, and on the lateral radiographs, the femoral component was aligned to be perpendicular to the beam [8,12]. Again, on the lateral view, the femoral condyles were superimposed, and the tibial implant was parallel to the horizontal plane (Figure 2). On misaligned radiographs, correctly positioned components may appear malpositioned or misaligned, leading to erroneous analyses [12].

The coronal alignment of the tibial implants was measured according to Hashemi et al. [13], with two circles drawn at a distance of 5 and 10 cm from the joint line and tangent to the medial and lateral cortex. The line passing between the midpoints of these circles and the line passing just underneath the tibial implant was used to measure coronal alignment. The sagittal alignment, on the other hand, was measured using the tibial proximal anatomical axis (TPAA) [14] (Figure 2). The TPAA was defined as the line connecting midpoints of two intracortical circles at 5 and 10 cm distal to the knee joint. Measurement of the slope was defined by the angle between the inclination line drawn just beneath the tibial implant and a line perpendicular to the aforementioned axis.

**Figure 3:**
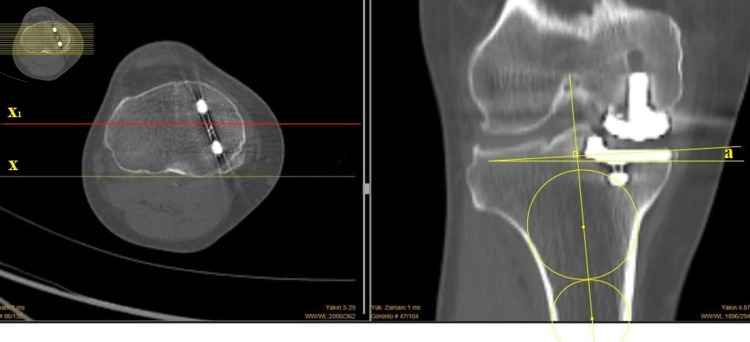
Coronal alignment on CT scans For coronal alignment on CT scans, the anatomical axis of the tibia was determined using two circles positioned 4-5 cm apart with the second circle placed as distally in the image as possible. The extended line connecting the two points at the center of the circles represented the longitudinal axis of the tibia. The coronal slices were set manually parallel to a line connecting the posterior tubercles of the tibia in the axial plane. At the level where the tibia was at its widest, coronal measurement of the implant was performed with the circle method, similar to the measurement performed on X-rays. CT: computed tomography

The CT scans were performed with a dual-source CT (Somatom® Definition Flash, Siemens Healthcare, Forchheim, Germany). Axial 1.5-mm slices were obtained and then multiplanar reconstruction was performed. In these planes, the anatomical axis of the tibia was again determined using two circles positioned at 4-5 cm apart with the second circle placed as distally in the image as possible. The extended line connecting the two points at the center of the circles represented the longitudinal axis of the tibia [15]. The coronal slices were set manually parallel to a line connecting the posterior tubercles of the tibia in the axial plane. In these reconstructed images, when approaching from posterior to anterior, at the level where the tibia was at its widest, coronal measurement of the implant was performed with the circle method described above (Figure 3).

**Figure 4:**
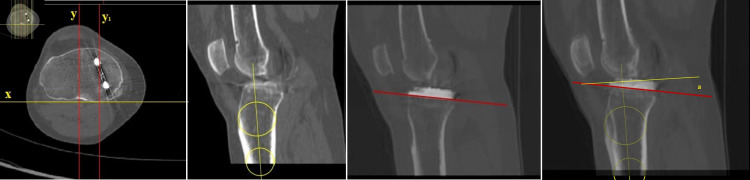
Sagittal alignment on CT scans At the level of the line connecting the posterior tubercles of the tibia, another line was drawn perpendicular to that. The sagittal slices were obtained in reference to this perpendicular line. The midsagittal slice that crosses through the intercondylar eminentia where the tibia is at its widest and the sagittal slice that crosses through the implant were overlapped. Then, sagittal measurements were performed with the circle method described in the text CT: computed tomography

In the axial images, at the level of the line connecting the posterior tubercles of the tibia, another line was drawn perpendicular to that. The sagittal slices were obtained in reference to this perpendicular line. In this reconstructed plane, the midsagittal slice that crosses through the intercondylar eminentia where the tibia is at its widest and the sagittal slice that crosses through the implant were overlapped. Then, sagittal measurements were performed with the circle method described above [11] (Figure 4). The tibial proximal anatomical axis was again used since it has previously shown a good correlation with X-rays [16]. It is also independent of variables such as age, gender, weight, and height and has a margin of error of ± 1 [11,13,14].

Data analysis

Direct radiographic and CT scan measurements were performed by two independent observers (an orthopedic surgeon and a radiologist) on two separate occasions. Measurements were performed using the angle measurement feature found in the local picture archiving and communication system (PACS). The observers were aware of the scope of the study but were blinded from each other. Intra- and inter-observer reliability was calculated and only the mean values of the obtained measurements were used in the final analysis. A correlation analysis was performed between values obtained from plain radiographs and those obtained from CT scans.

Statistical analysis

Statistical analysis was carried out using SPSS Statistics 22.0 (IBM, Armonk, NY) and Stata version 15.0 (StataCorp LLC, College Station, TX) software. In the descriptive analyses, categorical variables are stated as numbers (n) and percentages (%), and continuous variables as means ± standard deviations (SD) and median (minimum-maximum) values. Inter- and intra-observer reliability was analyzed and an intraclass correlation coefficient (ICC) was calculated. The ICC was also used to describe the correlation between the mean values of the angles measured on the CT scan and on the X-ray. All values were evaluated with a normality test. Pearson correlation was used for normally distributed values while a Spearman correlation test was used for values that were not normally distributed. To assess differences in the intra-observer variability between the X-ray and CT scan values, we used the Wilcoxon matched-pairs test. We also calculated the standard deviation for all angles and their 95% confidence intervals (CI). Linear regression between the mean X-ray values and the CT values yielded a prediction expression for the conversion of X-ray measurements to CT values. A p-value of <0.05 was considered statistically significant.

Reliability of measurement (margin of error) between an X-ray and a CT scan was compared using the typical error (TE) described by Hopkins [17]. This is an accepted measure used in assessing the reliability of measurement and is closely related to the limits of the agreement described by Bland and Altman [18]. We chose it because of its self-explanatory appearance; it shows the variation in the values of repeated measurements.

## Results

Sixty-one patients and 66 knees were analyzed for this study. The mean age of the cohort was 57.76 years, and 77.3% of the patients were female. The most frequently used implants were the mobile-bearing cementless version of the Oxford knee (Biomet Orthopedics, lnc, Warsaw, IN) and the fixed-bearing cemented ZUK knee (Zimmer Unicompartmental High Flex Knee System, Zimmer, Warsaw, IN). All descriptive and demographic data are presented in Table 1.

Intra-observer reliability was high among the two observers whether for X-ray or CT scan measurements (ICC: >0.900 for all planes). Inter-observer reliability, on the other hand, was different for coronal and sagittal plane measurements. Coronal plane measurements had lower ICC values on both X-rays and CT scans (ICC: 0.850 and 0.800 respectively), while reliability on the sagittal plane was higher (ICC: >0.900 for both). The TE (variation of repeated measurements) was lower for the sagittal plane values of both measurement modalities, signifying a lower margin of error. All data is shown in Table 2.

**Table 1:** Demographic and descriptive data SD: standard deviation

Variables	Values (n=66)
Age, years	
Mean ± SD	57.76 ± 6.624
Median (min-max)	56 (46-74)
Sex, n (%)	
Male	15 (22.7%)
Female	51 (77.3%)
Side, n (%)	
Right	38 (57.6%)
Left	28 (42.4%)
Implant, n (%)	
Oxford (Biomet)	41 (62.1%)
ZUK (Zimmer)	24 (36.4%)
Sled (Link)	1 (1.5%)

**Table 2:** Intra- and inter-observer reliability data *Variation of repeated measurements CT: computed tomography; ICC: intraclass correlation coefficient

		Direct radiograph measurement, n=66, ICC (95% CI)		CT scan measurement, n=66, ICC (95% CI)	
Coronal plane		Observer 1	Observer 2	Observer 1	Observer 2
	Inter-observer ratio	0.953 (0.924–0.971)	0.980 (0.967–0.988)	0.985 (0.975–0.991)	0.970 (0.951–0.982)
	Intra-observer ratio	0.850 (0.767–0.906)		0.800 (0.692–0.872)	
Sagittal plane		Observer 1	Observer 2	Observer 1	Observer 2
	Inter-observer ratio	0.920 (0.872–0.950)	0.973 (0.956–0.983)	0.976 (0.960–0.985)	0.985 (0.976–0.991)
	Intra-observer ratio	0.940 (0.904–0.963)		0.971 (0.954–0.982)	
		Typical error*			
Coronal plane inter-observer error		1.7		1.7	
Sagittal plane inter-observer error		1.1		0.7	

**Figure 5:**
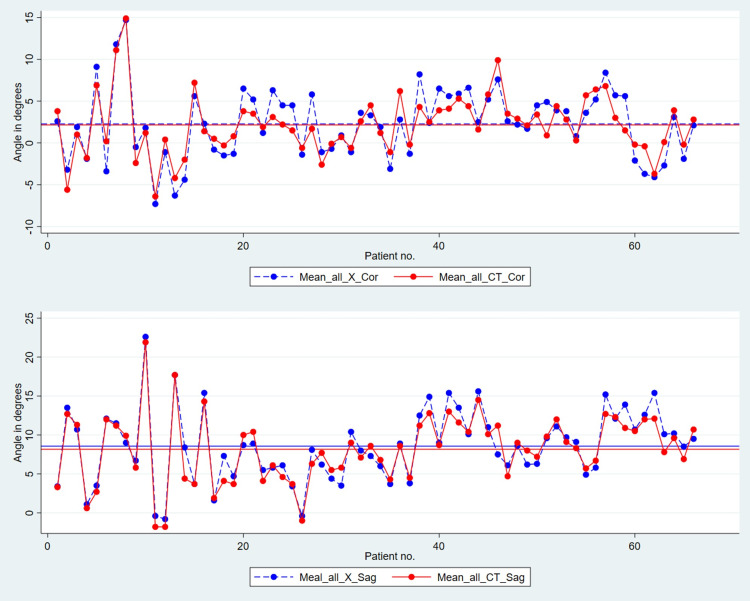
An overlay plot of overall mean X-ray and CT scan values measured by both observers The plot shows the correlation of the coronal (above) and sagittal alignment (below) on X-ray and CT. X-ray values are shown in blue dots and dotted lines while CT scan values are represented with red dots and full lines. The horizontal lines in the middle show the mean X-ray (dotted blue line) and CT values (full red line) values CT: computed tomography

There was a high correlation between radiographic measurements on X-ray and CT scans on both planes (Figure 5). All data is presented in Table 3. The mean difference between the measurements (X-ray and CT scan) was 0.1 ± 1.9 and 0.4 ± 1.4 degrees for coronal and sagittal planes, respectively (Table 4).

**Table 3:** Correlation coefficients between the measurement modalities *Pearson correlation CT: computed tomography

	Correlation between direct radiographic measurements and CT scans	
	Correlation coefficient R*	P-value (significant)
On the coronal plane	0.89	<0.001
On the sagittal plane	0.952	<0.001

**Table 4:** Relationship between the mean values of both modalities and margin of error *Wilcoxon matched-pairs test CT: computed tomography

	Mean values of measurements and margin of error between measuring modalities	
	CT scan Observer 1 + Observer 2	X-ray Observer 1 + Observer 2
Coronal plane	2.1545 ± 3.6340	2.2727 ± 4.2380
	(95% CI: 1.261–3.048)	(95% CI: 1.231–3.315)
Range	-6.4–14.9	-7.3–14.7
Difference	0.1 ± 1.9	
	(95% CI: -0.3580–0.5944)	
	-3.60–4.10	
	P=0.622*	
Sagittal plane	8.1697 ± 4.3841	8.5621 ± 4.5983
	(95% CI: 7.092–9.247)	(95% CI: 7.432–9.693)
Range	-1.8–21.9	-0.8–22.6
Difference	0.4 ± 1.4	
	(95% CI: 0.0458–0.7390)	
	-3.70–4.00	
	P=0.273*	

We also computed predictive formulas relating the measurement on X-ray to that on CT scans:

(Coronal) value on CT scan = 0.763 x (value on X-ray) + 0.420 r2 = 0.792

(Sagittal) value on CT scan = 0.908 x (value on X-ray) + 0.400 r2 = 0.906

## Discussion

This study shows that direct radiographs are a relatively reliable means to assess change in the alignment of the tibial component after a UKR procedure. Measurements of the sagittal plane (i.e., posterior tibial slope) on X-rays highly correlated with real values measured on CT scans and has a lower margin of error (± 1.4).

The threshold for revision after UKR has historically been higher than in total knee replacements [19]. Two of the well-known reasons for this have been the asymptomatic radiolucent and the ill-defined aseptic loosening [8]. While radiolucent lines are a frequent phenomenon in cemented UKR, they are relatively benign. Subsidence and early migration have often been defined as aseptic loosening leading to early and unnecessary revisions. Although each manufacturer has its own limits of normality, tibial component alignment on the coronal plane is generally accepted to be between -5 and 5 degrees while sagittal alignment is considered normal between 5-7 degrees [8,20]. Measuring degrees on a direct radiograph can be difficult and minimal rotation could lead to angular errors ranging from ± 13 to ± 1 degree. Hudek et al. [11], in their study with X-rays and MRI, reported that sometimes rotation on X-rays could lead to errors in measurements of up to 23 degrees. To minimize these errors, standard radiographs with minimal rotation are used for measurements. Our study shows that standard radiographs provide reliable and relatively accurate measurements. The angular values obtained from these measurements are very close or at least highly correlate to the real values (± 1.9 and ± 1.4 degrees for coronal and sagittal planes, respectively). This is especially true for sagittal measurements, since obtaining a true anteroposterior X-ray of an unicondylar knee implant can sometimes be tricky and minor rotation can be overlooked, leading to erroneous results.

Migration and subsidence regarding the tibial component of UKR has been the topic of many previous studies. The current literature supports the view that early postoperative subsidence, especially in cementless cases, is acceptable and may be inevitable in the first months [9,10]. This subsidence, which is both on the coronal and sagittal plane, tends to stop or diminish in the years following surgery, and in cementless cases, it is widely believed to be due to the bedding-in phenomenon. Cemented cases, despite not requiring a bedding-in period, have also been reported to be prone to early postoperative subsidence [9,21,22]. Measuring subsidence and the resulting change in the coronal and sagittal plane is not always an easy process. Currently, the gold standard procedure is the RSA, which consists of the intraoperative insertion of extra tantalum landmarks and standard views taken in a custom cage [23,24]. Although it offers the opportunity to analyze even minimal migratory movements and only requires a small number of subjects to achieve significance in research studies, it is a relatively expensive procedure and requires special equipment not always present in all orthopedic centers. CT scans are easier to obtain and give a precise measurement of the current coronal and sagittal alignment [25]. However, in order to detect possible subsidence or migration, scan repetition and therefore extra exposure to radiation is needed. On the other hand, plain radiographs are simple to perform, relatively harmless, and easily repeatable, but their results are often dependent on the observer making the measurements and it is difficult to obtain well-aligned views. Previous studies have shown good reliability with plain radiographs [11,13,14], and our study only confirmed this. High reliability was found across the platforms (ICC: >900) with slightly lower values (ICC: 800-850) for coronal alignment measures. We believe this to be the result of minor rotational malalignments while taking the X-rays. Using calibrated X-rays and preferably under fluoroscopic guidance would yield better views, thereby leading to sounder measurements.

Our results have some limitations. All measurements were mostly performed on only two different implants, and despite these two implants being the most frequently used ones worldwide, we do not know whether these measurements and correlations are valid for other implants, cemented or not. An optimal X-ray may sometimes be difficult to obtain and several attempts may be required before getting a radiographic view on which accurate measurements can be performed. This can be overcome by using an image intensifier prior to the real X-ray. In most cases, this should lead to reliable measurements without the need for a CT and therefore excessive radiation. Our number of cases was relatively low. A higher number of X-rays and CT scans would have made our analysis more powerful. Another limitation of this study is the lack of axial rotational measurements.

## Conclusions

Our initial hypothesis regarding the correlation of measurements performed on plain radiographs was proven to be true. Although the measurements on plain radiographs were slightly different from the ones obtained from CT scans, the correlation between them was very strong. We also showed good inter and intra-observer reliability. Care should be taken when measuring the coronal alignment of the tibial implant on X-rays, since it is more frequently affected by rotational misalignment.
